# The world’s largest potato cryobank at the International Potato Center (CIP) – Status quo, protocol improvement through large-scale experiments and long-term viability monitoring

**DOI:** 10.3389/fpls.2022.1059817

**Published:** 2022-11-29

**Authors:** Rainer Vollmer, Rosalva Villagaray, Mario Castro, José Cárdenas, Sandra Pineda, Janeth Espirilla, Noelle Anglin, Dave Ellis, Vânia Cristina Rennó Azevedo

**Affiliations:** ^1^ Genebank, International Potato Center (CIP), Lima, Peru; ^2^ Small Grains and Potato Germplasm Research unit, US Department of Agriculture (USDA), Aberdeen, ID, United States

**Keywords:** potato, *Solanum*, cryopreservation, cryoconservation, cryobank, viability, recovery, PVS2

## Abstract

Long-term conservation of Plant Genetic Resources (PGR) is a key priority for guaranteeing food security and sustainability of agricultural systems for current and future generations. The need for the secure conservation of genetic resources collections *ex situ* is critical, due to rapid and extreme climatic changes which are threatening and reducing biodiversity in their natural environments. The International Potato Center (CIP) conserves one of the most complete and diverse genetic resources collections of potato, with more than 7500 accessions composed of 4900 cultivated potato and 2600 potato wild relative accessions. The clonal conservation of cultivated potato, principally landraces, through *in vitro* or field collections is indispensable to maintain fixed allelic states, yet it is costly and labor-intensive. Cryopreservation, the conservation of biological samples in liquid nitrogen (-196°C), is considered the most reliable and cost-efficient long-term *ex-situ* conservation method for clonal crops. Over the last decade, CIP has built one of the largest potato cryobanks worldwide, cyopreserving more than 4000 cultivated potato accessions which represents 84% of the total cultivated potato collection currently conserved at CIP. In approximately, four years the entire potato collection will be cryopreserved. The development of an applied, robust cryopreservation protocol for potato, serves as a model for other clonally maintained crop collections. The CIP cryobank designs experiments with a high number of genetically diverse genotypes (70-100 accessions, seven cultivated species), to obtain reliable results that can be extrapolated over the collection as genotypes can often respond variably to the same applied conditions. Unlike most published reports on cryopreservation of plants, these large-scale experiments on potato are unique as they examine the acclimatization process of *in vitro* plants prior to, as well as during cryopreservation on up to ten times the number of genotypes conventionally reported in the published literature. As a result, an operational cryopreservation protocol for potato has advanced that works well across diverse potato accessions, not only with reduced processing time and costs, but also with an increased average full-plant recovery rate from 58% to 73% (+LN) for routine cryopreservation. The present article describes the composition of CIP’s cryobank, the cryopreservation protocol, methodology for the dynamic improvement of the operational protocol, as well as data collected on regeneration from long term cryopreserved potatoes.

## Introduction

Potato is the third most important staple crop worldwide, with a total production of 437x10^6^ tons on 20.7x10^6^ hectares, and an average yield of 20.9 tons/hectare (Devaux et al, 2014; FAOSTAT, 2022). Potatoes are rich in carbohydrates, protein, vitamin C, vitamin B6, magnesium, potassium, and fiber and frequently used as a primary nutrient source in countries where people cannot afford high energy diets (Zaheer and Akhtar, 2016).

The agrobiodiversity of important food crops is predominately conserved as dried botanical seed at cold temperatures. However, for 50% of the ten most important crops for human livelihoods (Goldshein, 2011), potato, cassava, sweet potato, yam, and banana, conserving the unique allelic combination in an individual (clone) is desired and this cannot be conserved through botanical seed. Thus, different conservation strategies, such as *in situ* conservation, conservation as an *ex situ* collection in the field or greenhouse, conservation of collections of vegetative propagules (e.g. tuber collections), *in vitro* conservation, or cryopreservation are utilized. At the International Potato Center, potatoes were mostly maintained as *ex situ* field collections of tubers which needed to be regenerated annually; however, in the 1980’s an *in vitro* laboratory was established, and potato accessions were introduced and conserved *in vitro*. As cultivated potato is a clonally propagated crop, it is principally conserved in genebanks as tubers (at 4°C) or *in vitro* collections (at 7-22°C).

While the tubers have the advantage that stored germplasm can be directly planted in the field, infection by pathogens or insects, short storage periods, along with challenges due to space, cost and labor needed for regeneration and maintenance, are limiting factors for long-term conservation of tubers. In contrast, *in vitro* potato germplasm collections can be conserved as plants in test tubes, which can be maintained long-term as pathogen-free material, facilitating distribution of the collections for use. Although the *in vitro* storage period can be extended to 2-3 years between subcultures or propagation cycles, the plant material needs to be renewed periodically and any handling of the genebank material exposes the collection to the risk of human error (mix up of accessions), fungi or bacteria contamination, or somaclonal variation sometimes produced from tissue culture. As the renewal process occurs repeatedly, the probability of changes, due to human error or biology of the conserved material, can add up over time (Anglin et al., 2021). Many of the above-mentioned disadvantages can be lessened, mitigated, or eliminated using cryopreservation for clonal crop maintenance. This is one of the reasons the CIP genebank chose to begin a cryopreservation program.

Cryopreservation is defined as the conservation of biological material at ultra-low temperatures, generally in the liquid (-196°C) or vapor phase of liquid nitrogen (LN) and is considered a cost- and space-efficient long-term conservation method for clonal crops (Vollmer et al., 2021). *“Cryopreserved plant materials can theoretically remain alive for centuries, after which they can be removed from the frozen conditions and regenerated into healthy, growing plants”* (Food and Agriculture Organization (FAO), 2021). The genetic and morphological stability of cryopreserved material was confirmed in multiplies studies with diverse species, e.g. *Vanilla planifolia*, *Solanum tuberosum, Hedeoma todsenii*, *Passiflora pohlii*, Rubus *grabowskii, Malus*, etc. (Volk et al., 2008; Castillo et al., 2010; Merhy et al., 2014; Li et al., 2017; Pence et al., 2017; Gonzáles-Arnao et al, 2022)

In the late 1990s, CIP initiated research into cryobanking as a long-term conservation method for potato and other clonal crops. Many factors contributed to the decision to begin cryobanking and the reasons included the long-term security and stability of the collection, the reduction of annual maintenance costs for *in vitro* conservation of potato and the ongoing challenge of needing skilled staff to phytosanitary clean and conserve a collection of 4850 potato *in vitro* accessions. While the cost of placing a sample into cryo is high (~400 US$/acc.), the annual maintenance cost once it is in cryo is ~7 US$/acc. (includes periodic replacement of cryotanks and a LN generator after 20 years of use) which overall is much lower than the annual cost of routine *in vitro* conservation (~65 US$/acc.).

Cryopreservation protocols for plants are typically developed and optimized with a small number of accessions, with the assumption that the results obtained can be successfully applied to large cryo-collections of hundreds or thousands of diverse genotypes. This is usually not the case because germplasm collections consist of high diversity and numerous different species. Therefore, variation in responses to virtually any manipulation exists, leading to the need for tailoring of protocols to adjust for variability in responses. Thus, at CIP, cryopreservation experiments are set up with >70 accessions including all cultivated species to help ensure robust application of results (Vollmer et al., 2018). Further, clear minimum *a priori* criteria have been established for accessions to qualify undergoing cryopreservation (viability rate, phytosanitary state, verification of genetic identity, representation of diversity, etc.), as well as the application of high-quality standards for all steps of the cryopreservation process (Reed, 2008; Food and Agriculture Organization (FAO), 2014; Vollmer et al., 2016).

There are very few reports in the literature on large-scale, long-term monitoring of viability of cryopreserved plant material in cryobanks (Vollmer et al., 2017). For such long-term monitoring of viability of a cryo collection through time, one needs to have a long-term vision of more than 100 years (more than the lifetime of a single scientist), and therefore, the number of samples placed into cryopreservation needs to be large enough to permit periodic future assessment of the viability of cryobanked accessions, without compromising the cryobank stock for long-term genebank conservation.

Lessons learned through the experience of operational-scale potato cryopreservation and adjustments made to the protocol to improve the efficiency of cryopreservation over time have produced a rigorous quality management system (QMS) which includes a viability monitoring program with extensive data collected on recovery and survivability of potato accessions after cryopreservation. Details from experiments and knowledge gained are presented to benefit others cryopreserving large plant genetic resources collections.

## Materials and methods

### Routine cryopreservation

Nodal stem segments from *in vitro* potato plants maintained in CIP’s *in vitro* medium-term collection (7 ± 2°C with a 16h photoperiod and light intensity of 5-20 µmol.m^-2^.s^-1^) were maintained under active growth conditions and subcultured in 25x150 mm glass test tubes every 3-4 weeks (genotype-dependent), with 4-5 explants per tube, on solid Murashige and Skoog medium (MS) [Murashige and Skoog, 1962], supplemented with standard vitamins (MSP09, Caisson Laboratories, East Smithfield, PA), 25 g.L^−1^ of sucrose and 3.0 g.L^−1^ of Phytagel™ (P8169, Merck Sigma-Aldrich^®^, St. Louis, MO). The pH of the culture medium was adjusted to 5.60 ± 0.02 with NaOH or KOH (1-2 M) and HCl (1-2 M), prior to autoclaving at 121°C for 20 min. The multiplied plant material was incubated at 20 ± 2°C, with a 16h photoperiod and light intensity of 80–100 μmol.m^−2^.s^−1^ (fluorescent tubes, 36W, cool day light). *In vitro* plants were multiplied from one single tube (medium-term storage), to three, six, and fourteen tubes, in three subsequent subculture cycles within a period of 9-12 weeks.

**Figure 1:**
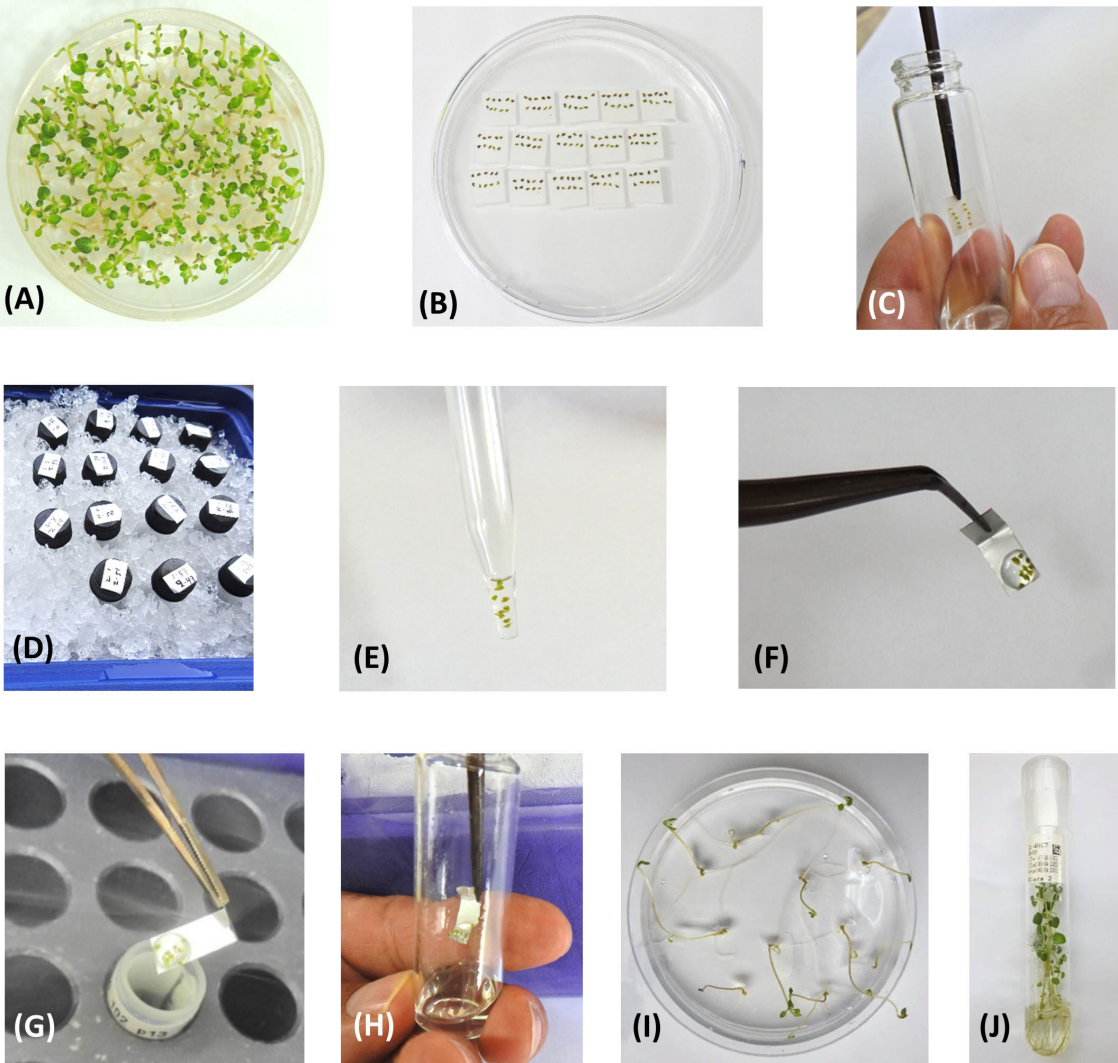
Steps in the CIP potato PVS2-droplet vitrification cryopreservation protocol. **(A)** *In vitro* potato plants grown from uninodal axillary stem segments after incubation for one week at 20 ± 2°C (“bud breaking”), followed by a cold acclimatization period of 2-3 weeks at 7 ± 1°C. Plants shown are ready for shoot tip excision. **(B)** Excised shoot tips (0.8-1.2 mm of size; genotype-dependent), placed on small filter papers, prior to cryoprotection treatment. **(C)** Introduction of shoot tips into vial containing 2 mL of Loading Solution (LS). Shoot tips are treated for 20 min in LS. **(D)** Treatment with Plant Vitrification Solution 2 (PVS2) [on ice, for 50 minutes]. **(E)** Ten shoot tips contained in the tip of a glass Pasteur pipette during the final minute in PVS2. **(F)** Shoot tips contained in a droplet of PVS2 placed onto a small aluminum foil strip (5x20 mm). **(G)** Aluminum strip is quickly plunged into liquid nitrogen (LN) (“Fast freezing”). **(H)** Thawing of shoot tips in 4 mL of Rewarming Solution (RS) at room temperature. Shoot tips are rewarmed for 20 minutes in RS. **(I)** Plants recovered from LN, 30 days after thawing. **(J)** *In vitro* plants 60 days after thawing, ready for further *in vitro* multiplication or direct transplant to the greenhouse.

The final, or fourth, multiplication cycle was performed in deep Petri dishes. In this case, uninodal stem segments were subcultured at a high density (80-90 explants per Petri dish), and incubated for one week under normal conditions (20 ± 2°C, 16h photoperiod, light intensity of 80–100 μmol.m^−2^.s^−1^), followed by a cold acclimatization period for two to three weeks (at 7 ± 2°C, 16h photoperiod, light intensity of 10-20 µmol.m^-2^.s^-1^), depending on the genotype-specific growth pattern of the accessions (**Figure 1A**).

Shoot tips, 0.8-1.2 mm long and 0.4-0.7 mm wide (genotype-dependent) containing 3-4 leaf primordia were excised from the cold-acclimated *in vitro* plants. Per accession, a total of 150 shoot tips were excised and placed on a small sterile piece of filter paper (size: 1x1 cm; 10 shoot tips per filter paper) that was supported on solid MS medium of the same composition as previously described, but supplemented with 2.8 g.L^−1^ of Phytagel™ (culture medium is contained in a standard Petri dish) [**Figure 1B**].

Each set of ten shoot tips was treated with 2 mL of Loading Solution (LS) at room temperature (20-25°C) for 20 minutes (**Figure 1C**). LS is composed of liquid MS medium (MSP09, Caisson Lab.), supplemented with 2.0 M glycerol (G5516, Merck Sigma-Aldrich^®^) and 0.4 M sucrose. The pH of the LS was adjusted to 5.80 ± 0.02. Loading solution, Plant Vitrification Solution 2 (PVS2), and Rewarming Solution (RS) were prepared with milli-Q water, while for culture media deionized-distilled water was used. Treatments with LS, PVS2, and RS were performed in sterile 15 mL glass screw top test tubes. The time that each treatment ends was noted on small labels and stuck onto the test tube caps.

After the loading phase, LS was removed from the test tube with a Pasteur pipette and replaced with 2 mL of pre-chilled PVS2 (0°C). Shoot tips were treated for exactly 50 minutes with PVS2 (on ice) (**Figure 1D**). PVS2 is composed of liquid MS medium (MSP09, Caisson Lab.), supplemented with 3.28 M glycerol, 2.42 M ethylene glycol (324558, Merck Sigma-Aldrich^®^), 1.9 M dimethyl sulfoxide (D4540, Merck Sigma-Aldrich^®^), and 0.4 M sucrose. The pH was adjusted to 5.80 ± 0.02.

One minute before the PVS2 treatment has concluded, shoot tips were sucked up with a Pasteur pipette (**Figure 1E**) and placed in a small droplet (20-25 µl) of pre-chilled PVS2, on the non-folded end of a small sterile aluminum foil strip (5 x 20 mm) [heavy duty quality, Boardwalk^®^ BWK 7136, Essendant Co., Deerfield, IL]. The aluminum foil strip was previously folded at one end (fold of ~ 3-4 mm) to facilitate handling with forceps. The ten shoot tips were contained within one single droplet of uniform shape and volume (**Figure 1F**). The aluminum foil strip was quickly plunged (“fast freezing”) into LN (**Figure 1G**) in a 1.8-mL cryovial (internal thread Nunc™, Thermo Fisher Scientific™, Waltham, MA) contained in a freezing box (CoolBox™ 30 Systems, Biocision, Larkspur, CA). Each cryovial contained one aluminum foil strip with ten shoot tips.

30 of the 150 shoot tips (three cryovials) from each cryopreservation run were thawed after a minimum 24h in LN to obtain the initial viability or recovery rate. For thawing, the aluminum foil strip was taken out of the cryovial and quickly plunged into 4 mL of recovery solution (RS) composed of liquid MS medium, supplemented with 0.6 M sucrose (pH: 5.80 ± 0.02) [**Figure 1H**]. The shoot tips were rewarmed in RS at room temperature for 20 minutes.

Thawed shoot tips were recovered with a Pasteur pipette and placed for ~30 seconds on a stack of 2-3 sterile filter papers to remove excess RS, and then transferred slipping the filter paper with the shoot tips facing down onto filter papers (2.5x2.5 cm) supported on MS culture media (as previously described), in a standard Petri dish (10x150 mm). The culture medium was supplemented with 25 g.L^-1^ sucrose, 20 mL.L^-1^ coconut water (C5915, Merck Sigma-Aldrich^®^), 0.1 mg.L^-1^ gibberellic acid (G7645, Merck Sigma-Aldrich^®^), 0.4 mg.L^-1^ of kinetin (K0753, Merck Sigma-Aldrich^®^), and 2.8 g.L^-1^ of Phytagel. The pH was adjusted to 5.60 ± 0.02. Three filter papers, each containing ten shoot tips (total: 30 shoot tips/Petri dish) were contained in each Petri dish. Petri dishes were wrapped completely in aluminum foil and incubated in the dark at 20 ± 2°C for nine days. After nine days, the ten shoot tips contained on each filter paper were removed from the filter paper and transferred directly onto individual Petri dishes with fresh culture medium of the same composition (each Petri dish contained 10 shoot tips). Samples were incubated at 20 ± 2°C, under diffuse light conditions (covering the top of the Petri dishes with a sheet of aluminum foil), with a 16h photoperiod and light intensity of 80–100 μmol.m^−2^.s^−1^. After four days, the aluminum foil was removed, and the samples were incubated for 17 additional days under normal light conditions. Samples that developed into complete *in vitro* plants or showed the potential to do so (bud breaking) (**Figure 1I**), were transferred to 25x150 mm glass test tubes containing the same culture medium, with a maximum of five plants per tube. Samples were incubated in the same environmental conditions for 30 more days.

Full plant recovery was assessed 60 days after thawing (**Figure 1J**). A sample was considered as recovered when it developed into a complete normal looking *in vitro* plant, with a functional apex, elongated stem, leaf formation, and rooting. Plants coming from each of the three Petri dishes were assessed independently (three replicates of ten shoot tips per accession).

### Large-scale experiment – Effect of sucrose in recovery cycle

A set of 73 diverse potato landraces (**Table 1**) selected from the CIP genebank were cryopreserved with the above-described routine cryopreservation protocol (Vollmer et al., 2021). After thawing, shoot tips were placed on culture media with four different sucrose concentrations (0.07 M, 0.1 M, 0.2 M, and 0.3 M), in the dark, for nine days before they were transferred to culture medium with the standard sucrose concentration (0.07 M). As a control treatment, the previously used routine protocol for recovery was used, i.e. shoot tips were maintained on 0.3 M sucrose-rich medium for three days, and then transferred every three days to medium with a step-wise reduction in sucrose (0.2 M and then 0.1 M sucrose, total time period: nine days), before being transferred to culture media with standard sucrose concentration (0.07 M sucrose). More than 3000 potato accessions were cryopreserved between 2010 and 2019 applying the routine control treatment.

**Table 1:** List of 73 potato landraces that were used in the large-scale experiment study.

Accession identifier	ITPGRFA GLIS-DOIs	Species/subspecies	Country of origin
**Accession identifier**	**ITPGRFA GLIS-DOIs**	**Species/subspecies**	**Country of origin**
CIP 704178	10.18730/AAV5	*S. tuberosum* subsp. *tuberosum*	Chile
CIP 705068	10.18730/B40~	*S. tuberosum* subsp. *tuberosum*	Chile
CIP 706679	10.18730/CH3~	*S. tuberosum* subsp. *tuberosum*	Chile
CIP 706713	10.18730/CJ0S	*S. tuberosum* subsp. *andigenum*	Argentina
CIP 704087	10.18730/A8A=	*S. tuberosum* subsp. *andigenum*	Bolivia
CIP 704767	10.18730/AVFK	*S. tuberosum* subsp. *andigenum*	Bolivia
CIP 704980	10.18730/B1HW	*S. tuberosum* subsp. *andigenum*	Bolivia
CIP 705127	10.18730/B5RF	*S. tuberosum* subsp. *andigenum*	Colombia
CIP 704057	10.18730/A7G9	*S. tuberosum* subsp. *andigenum*	Ecuador
CIP 705352	10.18730/BC0Y	*S. tuberosum* subsp. *andigenum*	Ecuador
CIP 705407	10.18730/BDF3	*S. tuberosum* subsp. *andigenum*	Mexico
CIP 700921	10.18730/91RP	*S. tuberosum* subsp. *andigenum*	Peru
CIP 701561	10.18730/96BN	*S. tuberosum* subsp. *andigenum*	Peru
CIP 702152	10.18730/9ATG	*S. tuberosum* subsp. *andigenum*	Peru
CIP 702190	10.18730/9B1Q	*S. tuberosum* subsp. *andigenum*	Peru
CIP 702439	10.18730/9D9N	*S. tuberosum* subsp. *andigenum*	Peru
CIP 702343	10.18730/9CJ=	*S. tuberosum* subsp. *andigenum*	Peru
CIP 703971	10.18730/A4X0	*S. tuberosum* subsp. *andigenum*	Peru
CIP 704406	10.18730/AGR9	*S. tuberosum* subsp. *andigenum*	Peru
CIP 704501	10.18730/AKKT	*S. tuberosum* subsp. *andigenum*	Peru
CIP 704541	10.18730/AMRT	*S. tuberosum* subsp. *andigenum*	Peru
CIP 703244	10.18730/9J9~	*S. stenotomum* subsp. *goniocalyx*	Bolivia
CIP 701611	10.18730/96W1	*S. stenotomum* subsp. *goniocalyx*	Peru
CIP 702961	10.18730/9H2Z	*S. stenotomum* subsp. *goniocalyx*	Peru
CIP 703282	10.18730/9K3P	*S. stenotomum* subsp. *goniocalyx*	Peru
CIP 703352	10.18730/9MV4	*S. stenotomum* subsp. *goniocalyx*	Peru
CIP 703777	10.18730/9ZNH	*S. stenotomum* subsp. *goniocalyx*	Peru
CIP 704243	10.18730/ACJQ	*S. stenotomum* subsp. *goniocalyx*	Peru
CIP 705458	10.18730/BEYD	*S. stenotomum* subsp. *goniocalyx*	Peru
CIP 705575	10.18730/BJGG	*S. stenotomum* subsp. *goniocalyx*	Peru
CIP 706036	10.18730/BZW0	*S. stenotomum* subsp. *goniocalyx*	Peru
CIP 702287	10.18730/9C0H	*S. stenotomum* subsp. *stenotomum*	Bolivia
CIP 702587	10.18730/9EQY	*S. stenotomum* subsp. *stenotomum*	Bolivia
CIP 703473	10.18730/9Q98	*S. stenotomum* subsp. *stenotomum*	Bolivia
CIP 704141	10.18730/A9T9	*S. stenotomum* subsp. *stenotomum*	Bolivia
CIP 704771	10.18730/AVKQ	*S. stenotomum* subsp. *stenotomum*	Bolivia
CIP 705952	10.18730/BXK1	*S. stenotomum* subsp. *stenotomum*	Bolivia
CIP 706250	10.18730/C5G*	*S. stenotomum* subsp. *stenotomum*	Bolivia
CIP 706845	10.18730/CNTU	*S. stenotomum* subsp. *stenotomum*	Bolivia
CIP 700362	10.18730/8X4P	*S. stenotomum* subsp. *stenotomum*	Peru
CIP 701676	10.18730/97HP	*S. stenotomum* subsp. *stenotomum*	Peru
CIP 702353	10.18730/9CM0	*S. stenotomum* subsp. *stenotomum*	Peru
CIP 702834	10.18730/9G63	*S. stenotomum* subsp. *stenotomum*	Peru
CIP 703288	10.18730/9K9W	*S. stenotomum* subsp. *stenotomum*	Peru
CIP 703312	10.18730/9KWA	*S. stenotomum* subsp. *stenotomum*	Peru
CIP 703314	10.18730/9KYC	*S. stenotomum* subsp. *stenotomum*	Peru
CIP 703318	10.18730/9M2G	*S. stenotomum* subsp. *stenotomum*	Peru
CIP 703709	10.18730/9XRY	*S. stenotomum* subsp. *stenotomum*	Peru
CIP 704043	10.18730/A72*	*S. stenotomum* subsp. *stenotomum*	Peru
CIP 705477	10.18730/BFGZ	*S. stenotomum* subsp. *stenotomum*	Peru
CIP 705569	10.18730/BJBB	*S. stenotomum* subsp. *stenotomum*	Peru
CIP 703294	10.18730/9KD*	*S. phureja*	Colombia
CIP 703506	10.18730/9R4=	*S. phureja*	Colombia
CIP 705802	10.18730/BSFH	*S. phureja*	Colombia
CIP 704203	10.18730/ABKX	*S. phureja*	Ecuador
CIP 706764	10.18730/CKD~	*S. phureja*	Ecuador
CIP 706825	10.18730/CN8J	*S. phureja*	Ecuador
CIP 701025	10.18730/92PF	*S. phureja*	Peru
CIP 703654	10.18730/9W7J	*S. phureja*	Peru
CIP 704859	10.18730/AY0T	*S.* ×*chaucha*	Bolivia
CIP 702208	10.18730/9B8Y	*S.* ×*chaucha*	Peru
CIP 704047	10.18730/A76U	*S.* ×*chaucha*	Peru
CIP 707129	10.18730/CS5*	*S.* ×*chaucha*	Peru
CIP 707136	10.18730/CS9U	*S.* ×*chaucha*	Peru
CIP 706211	10.18730/C4FU	*S.* ×*ajanhuiri*	Bolivia
CIP 706213	10.18730/C4G0	*S.* ×*ajanhuiri*	Bolivia
CIP 703810	10.18730/A0J9	*S.* ×*ajanhuiri*	Peru
CIP 704234	10.18730/ACEK	*S.* ×*juzepczukii*	Bolivia
CIP 706777	10.18730/CKT9	*S.* ×*juzepczukii*	Bolivia
CIP 706050	10.18730/C09D	*S.* ×*juzepczukii*	Peru
CIP 706776	10.18730/CKS8	*S.* ×*curtilobum*	Bolivia
CIP 702455	10.18730/9DET	*S.* ×*curtilobum*	Peru

The table shows accessions identifier (CIP number), International Treaty for Plant Genetic Resources (ITPGRFA) Global Information System Digital Object Identifier (GLIS-DOI) of the accession, species/subspecies, and country of origin.

The experiment was set up as a completely randomized factorial design (73x5), and repeated three times, with a sample size of n=15 per repetition (three replicates of five shoot tips); hence, the average recovery rates shown in the figures are based on 45 shoot tips. As data was not distributed normally (Anderson-Darling test: p<0.005), nor any adequate data transformation could be applied (e.g. arcsine square root transformation) [Velleman and Hoaglin, 1981], the data was analyzed with the non-parametric Kruskal Wallis test for multiple samples (α=0.05). Data analysis was performed with SPC for Excel (V 6.0, BPI Consulting) and Minitab™ 17.1.0. Python software (V3.11).

### Long-term viability monitoring experiment

In 2013, a long-term viability monitoring experiment was initiated (completely randomized design), cryopreserving each year a higher number of shoot tips per accession (240) for 8-14 additional accessions annually than the routine protocol. After a minimum of 24 hours in LN, a sample of 30 shoot tips was thawed and recovered with the current available routine protocol. After 2, 4, and 8 years in LN additional these same samples (30 shoot tips each) were pulled out from LN and recovered with the same protocol for thawing and recovery that was used for the original sample to evaluate the effect of time on these accessions in cryopreservation. The long-term design of the experiment is to remove additional samples after 16, 32, and 64 years and process them as previously described. To date, 101 potato accessions have been included in this experiment, and the 2-year, 4-year and 8-year data is based on 76, 52 and 17 accessions, respectively. The experiment includes 40 of 45 accessions of CIP’s diverse mini-core collection which includes seven cultivated potato species. Data was analyzed with the Minitab™ 17.1.0.

## Results

### Composition of CIP’s potato cryobank – Routine cryopreservation

The potato cryobank at CIP currently holds 4086 accessions, belonging to seven cultivated potato species based on the taxonomy of Hawkes (1990), representing 83.7% of the *in vitro* collection and 83.0% of the total potato clonal collection in the CIP genebank. Most of the accessions are *S. tuberosum* subsp. *andigenum* (2797 acc.), which accounts for 68.4% of all cryobanked accessions, followed by *S. stenotomum* subsp. *stenotomum* (264 acc.), *S. phureja* (165 acc.), *S. tuberosum* subsp. *tuberosum* (151 acc.), *S.* ×*chaucha* (120 acc.), and *S. stenotomum* subsp. *goniocalyx* (90 acc.). The bitter potatoes, *S.* ×*curtilobum*, *S.* ×*ajanhuiri and S.* ×*juzepczukii*, have less representation in the cryobank compared to the other species, with only 7-27 acc. per species (**Table 2**).

**Table 2:** Composition of the potato cryobank of the International Potato Center (CIP), classified by species/subspecies (based on the taxonomy of Hawkes, 1990), ploidy level (PL), number of accessions (NoA), countries of origin, and average recovery rate (ARR) and range of recovery rates (RoRR).

Species/subspecies		PL	NoA	Countries of origin	ARR (%)	RoRR (%)
*S. tuberosum* subsp. *tuberosum* L.		4x	151	Chile (121); Argentina (9); Peru (3); Mexico (3); New Zealand (3); Colombia (2); Bhutan (2); India (2); Philippines (2); Russia (2); Venezuela (1); Guatemala (1)	63.8%	25.0 – 100.0%
*S. tuberosum* subsp. *andigenum* (Juz. & Bukasov) Hawkes		4x	2797	Peru (1892); Bolivia (317); Ecuador (224); Argentina (138); Colombia (130); Venezuela (32); Guatemala (27); Mexico (26); Bangladesh (5); Russia (5); Philippines (1)	64.0%	20.0 – 100.0%
*S. stenotomum* subsp. *stenotomum* (Juz. & Bukasov) Hawkes		2x	264	Peru (185); Bolivia (72); Colombia (2); Ecuador (2); Russia (2); Argentina (1)	59.2%	23.3 – 100.0%
*S. stenotomum* subsp. *goniocalyx* (Juz. & Bukasov) Hawkes		2x	90	Peru (87); Bolivia (1); Chile (1); Costa Rica (1)	60.8%	25.0 - 100.0%
*S.* ×*chaucha* Juz. & Bukasov		3x	120	Peru (89); Bolivia (23); Ecuador (8)	67.1%	26.7 – 100.0%
*S. phureja* Juz. & Bukasov		2x	165	Colombia (86); Ecuador (65); Peru (12); Russia (2)	58.3%	26.0 – 100.0%
*S.* ×*ajanhuiri* Juz. & Bukasov		2x	11	Bolivia (10); Peru (1)	54.4%	25.0 – 83.3%
*S.* ×*juzepczukii* Juz.		3x	27	Bolivia (14); Peru (12); Argentina (1)	58.6%	23.3 – 90.0%
*S.* ×*curtilobum* Juz. & Bukasov		5x	7	Peru (5); Bolivia (1); Argentina (1)	59.1%	30.0 – 76.7%
Unclassified landraces		?	125	Peru (73); Bangladesh (12); Bolivia (11); Ecuador (11); Sweden (7); Argentina (4); Bhutan (3); Guatemala (2); Colombia (1); New Zealand (1);	62.3%	25.0 – 100.0%
Improved cultivars		4x	304	Peru (117); United Kingdom (14); United States of America (23); Mexico (20); Colombia (16); India (16); Czech Republic (13); Netherlands (12); Argentina (10); Germany (9); Poland (8); Chile (7); Ecuador (7); Brazil (6); Russia (3); Rwanda (3); Bolivia (2); China (2); Japan (2); Kenya (2); Canada (2); South Korea (1); Philippines (1); Belgium (1); Cameroon (1); El Salvador (1); Turkey (1); Tanzania (1); Uzbekistan (1); Vietnam (1); Democratic Republic of the Congo (1)	65.6%	28.3 – 100.0%
Research material		TBD	25	United Kingdom (10); Peru (7); Netherlands (5); Germany (2); Mexico (1)	64.2%	30.0 – 100.0%
**TOTAL**		**4086**		**63.5%**	

The number of accessions per country is indicated in parenthesis next to each country. TBD, To be determined.

Within the cryobank, 3757 of 4086 accessions are landraces (92.0%), that were collected or obtained from Andean countries, the primary center of diversity of cultivated and wild potato. 61% of the accessions are originally from Peru, followed by Bolivia (11%), Ecuador (8%), Colombia (6%), Argentina (4%) and Chile (3%). On the species-level, one or two countries of origin are predominant, e.g. 80% of the accessions of *S. tuberosum* subsp. *tuberosum* are derived from Chile, while for *S. tuberosum* susp. *andigenum*, *S. stenotomum* subsp. *stenotomum, S. stenotomum* subsp. *goniocalyx, S.* ×*chaucha*, and *S.* ×*curtilobum*, 68-97% of these accessions are originally from Peru. *S.* ×*ajanhuiri* has its origin mainly in Bolivia (91% of accessions), while *S.* ×*juzepczukiii* accessions were collected from Bolivia (52%) and Peru (44%). *S. phureja* has its primary center of diversity in Colombia and Ecuador, with 52% and 39% of the accessions coming from these two countries, respectively (**Table 2**).

The ploidy levels of the cryobanked potato species are 2x, 3x, 4x, and 5x. The diploid and pentaploid species showed a lower average recovery rate of 54.4 to 60.8%, compared to the tetraploid species (63.8 – 64.0%). Interestingly, a triploid *S. xchaucha* species showed the highest average recovery rate of all species (67.1%). The improved cultivars showed a high average full-plant recovery rate of 65.6%. When evaluating the recovery rate within each species, the accession-specific recovery rate determined to have a wide range from 20 to 100% (**Table 2**).

### Large scale experiment - Effect of sucrose concentration in recovery medium

**Figure 2:**
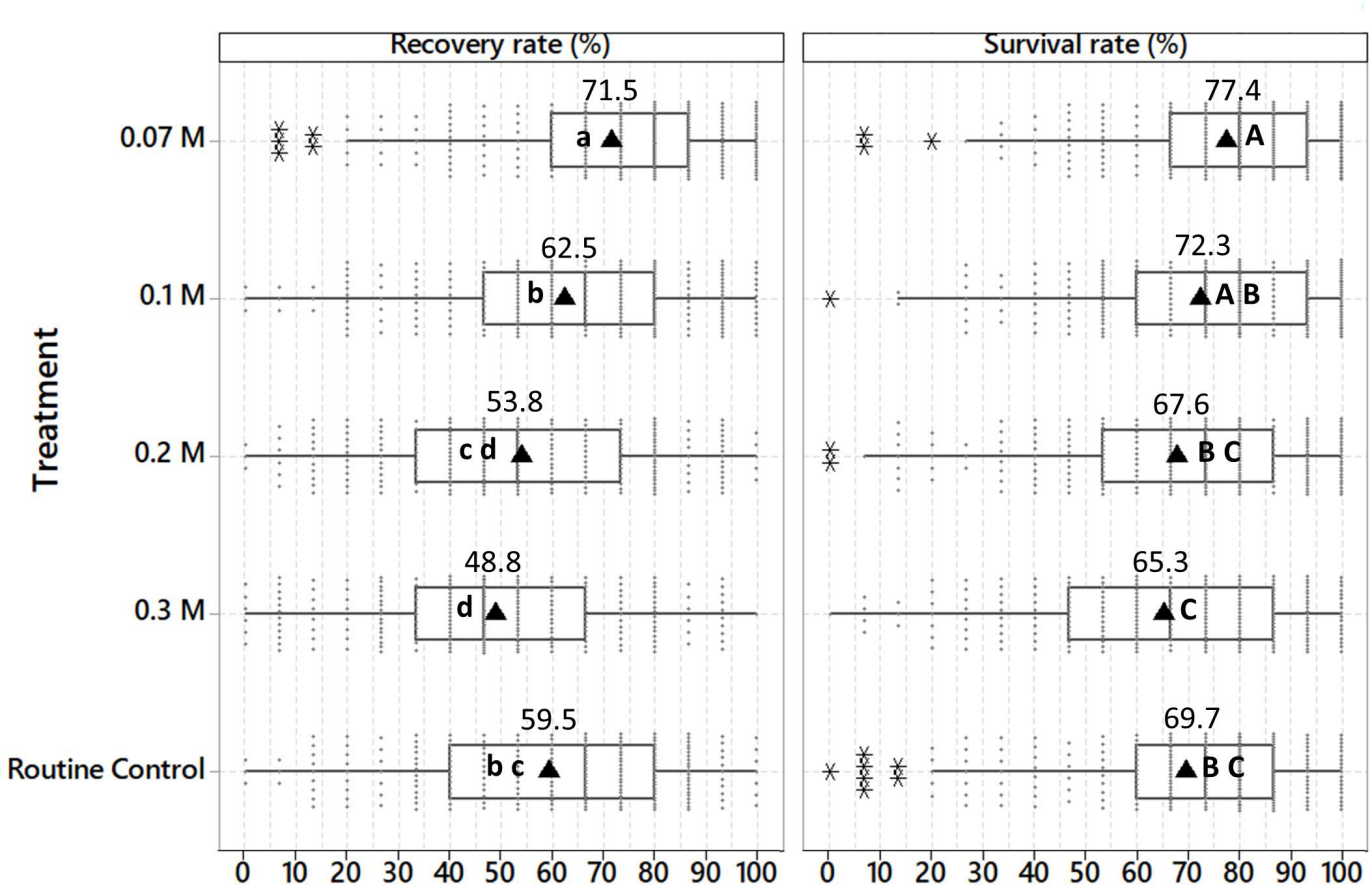
Average survival and recovery rates of 73 potato accessions cryopreserved with the PVS2-droplet vitrification method (+ LN), 60 days after thawing. A sample was considered as recovered when it developed into a complete normal looking *in vitro* plant, with a functional apex, elongated stem, leaf formation, and rooting. Plants that showed only leaf formation, greening, deformation or hyperhydration were considered as survival only. Shoot tips were excised from cold-acclimated *in vitro* plants (at 7°C) and cryopreserved with the PVS2-droplet vitrification method. Thawed shoot tips were placed for 9 days in darkness on culture media with different sucrose concentrations (0.07 M, 0.1 M, 0.2 M, and 0.3 M) and then transferred to culture medium with standard sucrose concentration (0.07 M). After thawing the shoot tips of the routine control treatment were maintained for 3 days on each of the 0.3 M, 0.2 M, and 0.1 M sucrose-rich culture medium (total period of 9 days, in darkness.), and then transferred to culture medium with standard sucrose concentration (0.07 M). The experiment was repeated three times (n = 15 per repetition and treatment). Different lower-case and upper-case letters indicate significant differences for the multiple Kruskal Wallis test of recovery and survival rate, respectively (p < 0.05). Filled circle: individual values; black triangle: mean value; asterisk: outlier value.

Placing shoot tips post-thaw on recovery medium with the normal sucrose concentration (0.07 M) for nine days significantly increased the average survival rate (SR: 77.4%) and recovery rate (RR: 71.5%), compared to the routine control cryopreservation protocol used at CIP (SR: 69.7%, RR: 59.5%), involving a step-wise decrease in sucrose concentration (three days on 0.3 M, 0.2 M and 0.1 M, respectively). Samples showing green tissue were recorded as survived, and those that developed into complete normal looking *in vitro* plants were considered as recovered. A stable sucrose concentration of 0.1 M resulted in a significantly higher recovery rate (62.5%) compared to higher sucrose concentrations of 0.2 M (RR: 53.8%) and 0.3 M (RR: 48.8%). The differential between SR and RR was higher with sucrose concentrations of 0.2 M and 0.3 M (13.8-16.5%), compared to lower concentrations of 0.07 M, 0.1 M, or the routine control treatment (5.9-10.2%) [**Figure 2**]. As the experiment was performed with 73 accessions representing seven potato species and four different ploidy levels, this represents a sample size of 1.5% in relation to the size of the complete *in vitro* potato collection at CIP.

43 of 73 assessed accessions (59%) showed the highest RR on culture medium with a sucrose concentration of 0.07 M, while 12 and six of 73 accessions had its highest RR at a sucrose concentration of 0.1 M and the routine control treatment, respectively. Surprisingly, five of the 73 tested accessions had the highest RR on a sucrose concentration of 0.2 M, which is a 2.7-times higher sucrose concentration compared to the standard culture medium of potato (**Table 3**).

**Table 3:** Percent of 73 cryopreserved potato accessions showing the highest recovery rate on each of the different post-thaw sucrose concentrations 60 days after thawing.

	Sucrose concentration in recovery medium during the first nine days of the recovery cycle							
	0.3 M	0.2 M	0.1 M	0.07 M	RC	0.07 M or 0.1 M	0.07 M or RC	0.1 M or RC
Number of accessions	0	5	12	43	6	2	2	3
Percent	0%	7%	16%	59%	8%	3%	3%	4%

Seven accessions showed the highest recovery rate with more than one treatment (three columns to the right, i.e. 0.07 M/0.1 M, 0.07 M/RC, and 0.1 M/RC). The treatments are described in the caption of **Figure 1**. RC, Routine Control.

**Figure 3:**
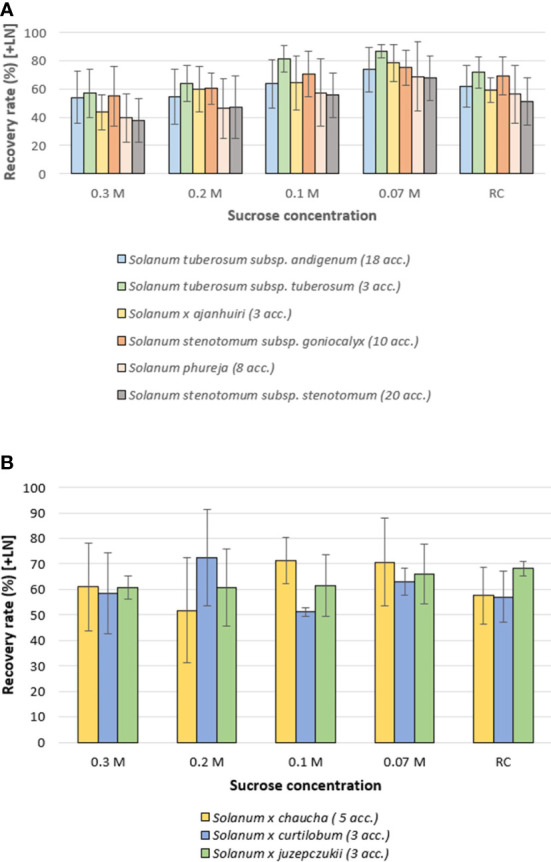
Average recovery rates of 73 cryopreserved potato accessions (+ LN), 60 days after thawing, classified by taxa. Thawed shoot tips were recovered on culture media with different sucrose concentrations (for details see caption **Figure 1**; RC, Routine Control). **(A)** Six species/subspecies of potato with similar patterns of recovery rate vs decreasing sucrose concentration in the recovery medium. **(B)** Three potato taxa that maintained stable recovery rates on culture medium with higher sucrose concentrations. Error bars: standard deviation.

Six species/subspecies, *S. tuberosum* subsp. *andigenum*, *S. tuberosum* subsp. *tuberosum*, *S. x ajanhuiri*, *S. stenotomum* subsp. *goniocalyx*, *S. stenotomum* subsp. *stenotomum* and *S. phureja*, showed a similar pattern, i.e. a pronounced decrease in RR as the sucrose concentration of the culture medium increased (0.07 M vs 0.1 M vs 0.2 M vs. 0.3 M). In absolute values of RR, the routine control showed a very similar pattern to the 0.1 M sucrose treatment (**Figure 3A**).

In contrast, *S. xchaucha*, *S. xcurtilobum* and *S. xjuzepczukii*, showed a relative stable RR with increasing sucrose concentration. In the case of *S. xcurtilobum* accessions, the highest RR was observed at a sucrose concentration of 0.2 M (**Figure 3B**).

**Figure 4:**
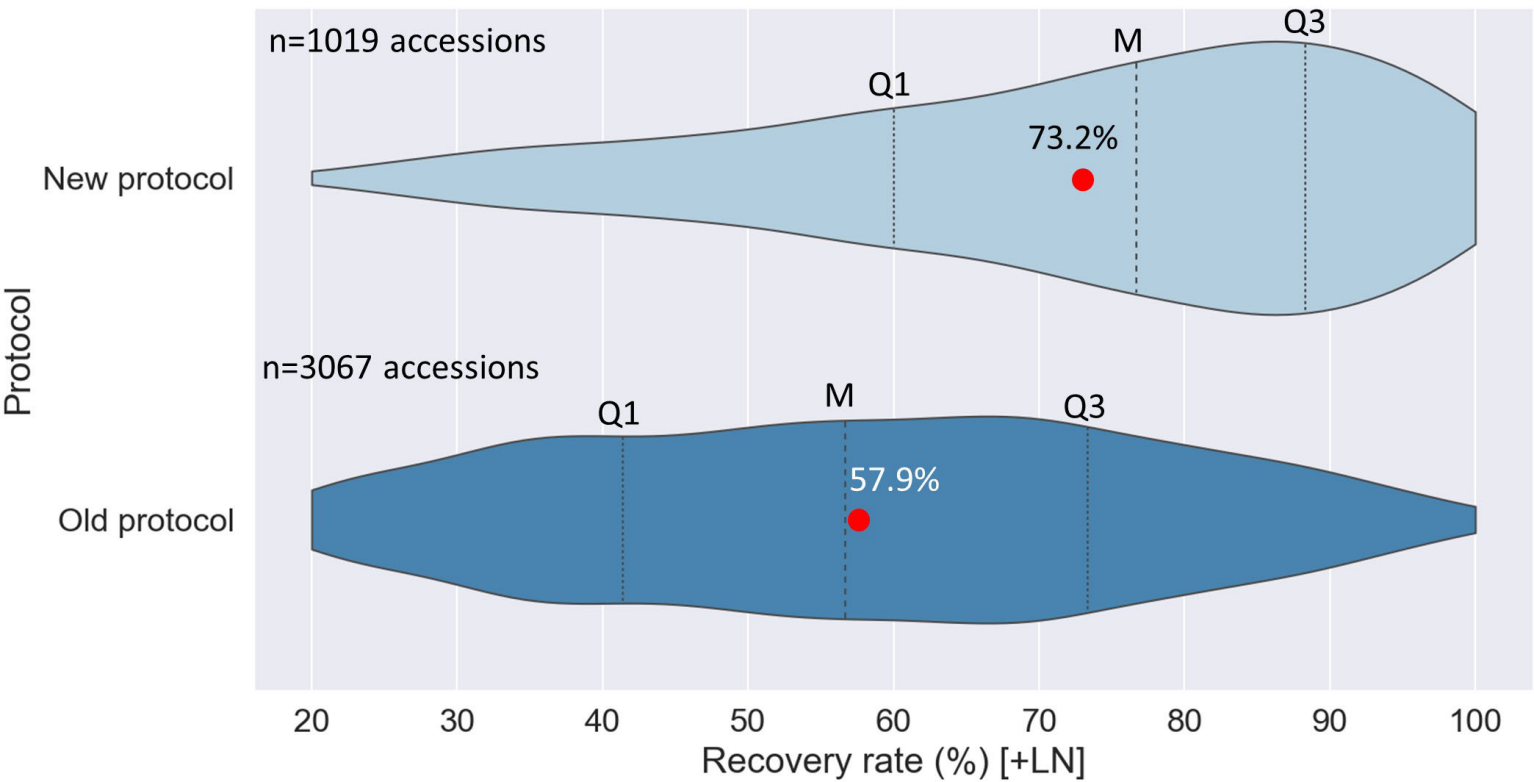
Violin plots of the distribution of recovery rate ranges for potato accessions cryopreserved with the old (3067 acc.) and new cryopreservation protocol (1019 acc.) [60 days after thawing]. Formerly, thawed/rewarmed potato shoot tips were recovered on culture media with a stepwise decrease in sucrose concentration, i.e., three days on 0.3 M sucrose-rich medium, three days on 0.2 M, three days on 0.1 M, all in darkness, then standard sucrose concentration of 0.07 M and normal light conditions. While for the new protocol, thawed/rewarmed shoot tips are placed for 9 days on culture medium with standard sucrose concentration (0.07 M; in darkness), and then incubated under normal light conditions. Q1: First quarter; Q3: Third quarter; M: median value; red circle: mean value.

The experimental results were confirmed during routine operations, with a substantial increase in the average RR from 57.9% (3067 accessions; routine control) to 73.2% (1019 accessions; 0.07 M sucrose). The mode of RR distribution, shifted from 60-70% to the 80-90% interval, i.e. the distribution is now skewed to the left with higher frequencies of RR close to the 80-100% ranges. In contrast, the distribution of RR of the routine control (3067 acc.), was close to normal, with 1694 and 1373 accessions showing recovery rates in the ranges of 20-60% and 61-100%, respectively (**Figure 4**).

### Long-term viability monitoring experiment

**Figure 5:**
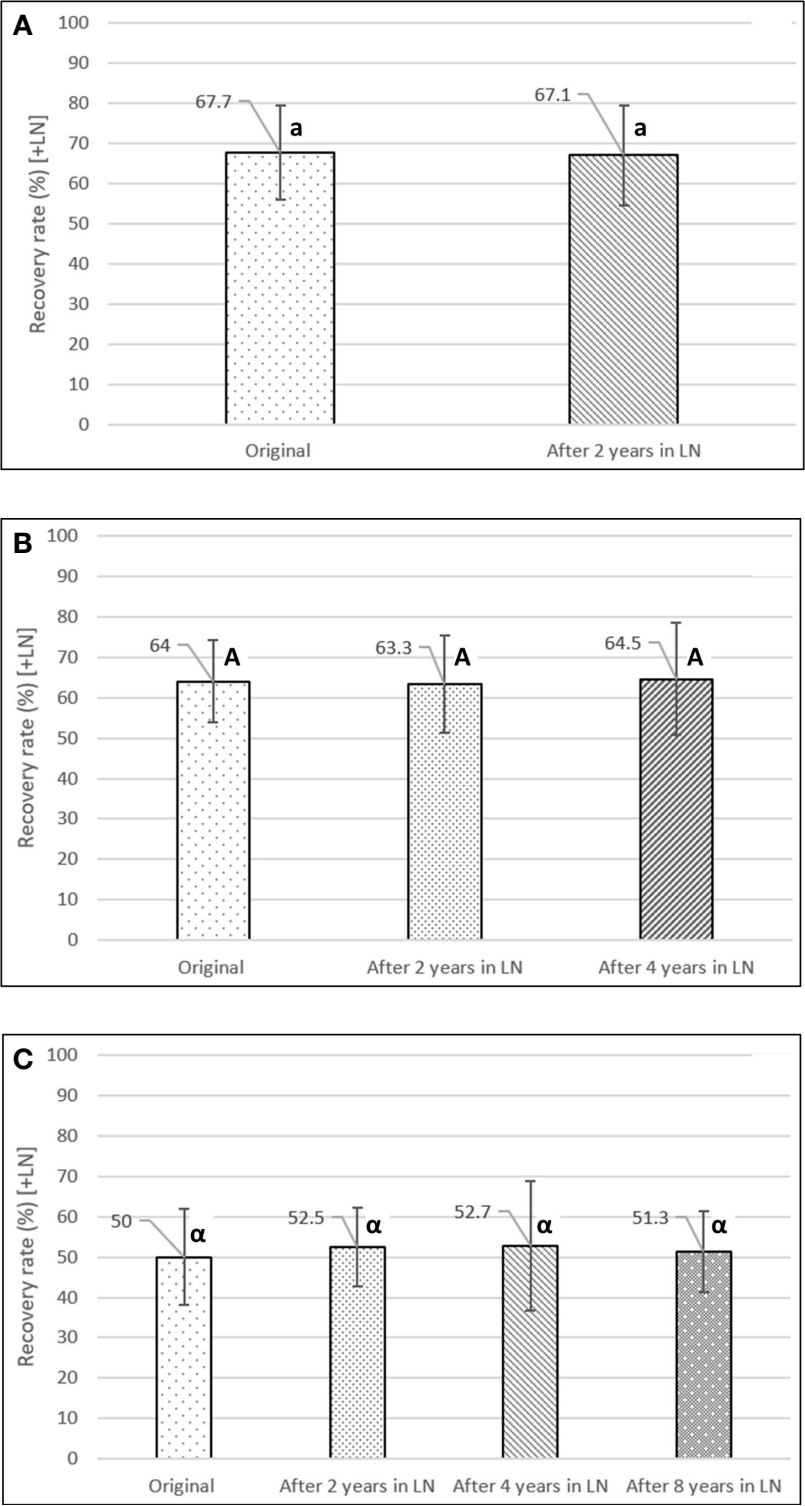
Long-term viability experiment of cryopreserved potato accessions. Full-plant recovery rates were assessed and compared after 0 (original), 2, 4, and 8 years in LN. **(A)** After two years in LN (76 accessions), **(B)** after four years in LN (52 accessions), **(C)** after eight years in LN (17 accessions). Different lower-, upper-case, and Greek letters indicate significant differences for the paired t-test, at a confidence level of 95%, for a hypothesized mean difference of less than 10% between years. Error bars: standard deviation.

After two, four, and eight years in LN, the accessions showed no significant differences in full-plant recovery rates for a hypothesized mean difference of less than 10% between years (paired t-test; confidence level of 95%). The two-year set (76 acc.) had an average recovery rate of 67.1% after two years in LN, compared to the original rate of 67.7% (after minimum 24h in LN) [**Figure 5A**]. The accessions of the four-year set (52 acc.) showed average recovery rates of 63.3% and 64.5%, after two and four years in LN, compared to the original rate of 64.0% (**Figure 5B**). Finally, after eight years in LN (17 acc.), the accessions showed a stable average recovery rate of 51.3%, compared to the original recovery rate (50.0%), and the average rates after two (52.5%) and four years (52.7%) in LN (**Figure 5C**). The average recovery rate of the 8-year set was lower than the other two time periods, as during this earlier time period, the potato cryopreservation protocol was still in development.

## Discussion

To the best of the authors knowledge CIP conserves the largest cryo-collection in the world belonging to one single genus (*Solanum*). Over ten years, CIP has built up its potato cryobank with ~4100 potato accessions for long-term storage, with an average full-plant recovery rate of 63.5%. The improvement of the first phase of the post-thawing recovery cycle (reduction in sucrose concentration in the post-thaw recovery medium), resulted in a substantial increase of the average recovery rate of approximately 15%, which was confirmed during routine cryopreservation with >1000 potato accessions (seven species). These results suggest that the long-held dogma that has existed in plant cryopreservation that a high sucrose concentration was needed to reduce the osmotic shock coming off the high osmotic levels in PVS2 may not be needed in all plants and indeed, could be detrimental to survival as evidenced in potato. These data are important as they further confirm the importance of the need for further investigation and refinement of post-thaw treatments for plant cryopreservation success.

It would be interesting to apply similar post-thaw experiments to other important clonal crop collections, such as cassava, yam and sweetpotato, as little information is available regarding collection-wide responses to cryopreservation. A critical point is that a relatively modest sampling with a sample size of 1.5% of the total collection (> 72 acc.), yielded results that reliably could be extrapolated to the entire collection [**Figure 4**]. This could be critical for development of robust operational cryopreservation protocols for other large clonal collections such as the cassava collections at The International Center for Tropical Agriculture (CIAT) and The International Institute of Tropical Agriculture (IITA) with 6155 and 4359 cassava accessions, respectively (CIAT, 2022; IITA, 2022), sweetpotato (5054 acc.) at CIP and yam (5839 acc.) at IITA (Genebank Platform, 2022). Although crop specific responses to cryopreservation are expected, the results presented here with reduced sucrose in the recovery medium could benefit these other collections as well.

Large cryopreserved plant genetic resources collections, have been established in only a small number of organizations around the world, e.g. the National Center for Genetic Resources Preservation (US, Fort Collins, CO), International Musa Germplasm Transit Centre (Europe, Belgium, Leuven), Leibniz Institute of Plant Genetics and Crop Plant Research (IPK, Europe, Germany, Gatersleben), International Potato Center (South America, Peru, Lima), National Institute of Agrobiological Sciences (Asia, Japan, Tsukuba), and National Agrobiodiversity Center (Asia, South Korea, Suwon) [Jenderek and Reed, 2017; Panis et al., 2020; Vollmer et al., 2021; Leibniz Institute of Plant Genetics and Crop Plant Research (IPK), 2022]. The second-largest potato cryobank after CIP is located at IPK (1845 accessions).

Further, improvements in the post-thawing steps of the cryopreservation protocol, should increase the recovery rate of those potato accessions that were previously introduced into the cryobank using the higher sucrose recovery media which was the standard at the time. Accessions belonging to the *S. xcurtilobum* and *S. xjuzepczukii* taxa seem to prefer a modified recovery cycle with a higher sucrose concentration (**Figure 3B**; **Table 3**). Specially those cryobanks that already have a significant number of accessions cryopreserved, could prioritize research on improvements of the recovery cycle. This will not only improve the general recovery potential of its cryo-collection, but also reduce the number of cryovials required to be removed in future thawing events (e.g. viability monitoring, distribution).

Modifications of other post-thaw variables, like oxidative stress, growth regulators (auxins, cytokinins), and environmental conditions have the potential to further increase the recovery rate of cryobanked material. Independent variables resulting in a low significance level for the interaction between genotype x treatment could be prioritized in larger-scale applications. The strategy employed in the present study where randomized and replicated experiments were done with a higher number of accessions and followed up during routine cryopreservation provides a very workable solution for large germplam collections (**Figures 2** , **4**). As a side effect, valuable evaluation data for a large and diverse number of genotypes are obtained, which can be used for genetic (Ellis et al., 2018), physiological, or other studies.

Additionally, the transitory results of ongoing long-term viability monitoring experiments show that the post-thaw viability rate of cryopreserved potato accessions is stable after two, four, and eight years in liquid nitrogen demonstrating the effectiveness and security of cryopreserving clonal accessions.

## Data availability statement

The raw data supporting the conclusions of this article will be made available by the authors, without undue reservation.

## Author contributions

RVo and DE designed and directed the study. RVo managed the data analysis, prepared figures and tables and drafted the manuscript. RVi, MC, SP, and JC performed the cryopreservation experiments, long-term viability monitoring study, and routine cryopreservation work, and reviewed and edited the manuscript. JE assisted with drafting and editing the manuscript. DE, NA, and VR supervised, reviewed and edited the manuscript. All authors contributed to the article and approved the submitted version.

## Acknowledgments

The authors express thanks for the financial support to the German Agency for International Cooperation (GIZ), the Global Crop Diversity Trust (GCDT) and the CGIAR Cooperative Research Program for Genebanks without their continuous support this work would not have been possible. We also acknowledge the staff in the cryobank for their hard work and dedication to germplasm conservation without which none of this would be possible.

## Conflict of interest

The authors declare that the research was conducted in the absence of any commercial or financial relationships that could be construed as a potential conflict of interest.

## Publisher’s note

All claims expressed in this article are solely those of the authors and do not necessarily represent those of their affiliated organizations, or those of the publisher, the editors and the reviewers. Any product that may be evaluated in this article, or claim that may be made by its manufacturer, is not guaranteed or endorsed by the publisher.
